# Proteomic changes in the xylem sap of *Brassica napus* under cadmium stress and functional validation

**DOI:** 10.1186/s12870-019-1895-7

**Published:** 2019-06-26

**Authors:** Jin-Song Luo, Zhenhua Zhang

**Affiliations:** 1 Southern Regional Collaborative Innovation Center for Grain and Oil Crops in China, College of Resources and Environmental Sciences, Hunan Agricultural University, Changsha, China; 2 Hunan Provincial Key Laboratory of Farmland Pollution Control and Agricultural Resources Use, National Engineering Laboratory on Soil and Fertilizer Resources Efficient Utilization, Hunan Provincial Key Laboratory of Nutrition in Common University, Changsha, 410128 China

**Keywords:** *Brassica napus*, Cd stress, Label free, Proteomics, Xylem sap. Plant defensin

## Abstract

**Background:**

The xylem sap of vascular plants primarily transports water and mineral nutrients from the roots to the shoots and also transports heavy metals such as cadmium (Cd). Proteomic changes in xylem sap is an important mechanism for detoxifying Cd by plants. However, it is unclear how proteins in xylem sap respond to Cd. Here, we investigated the effects of Cd stress on the xylem sap proteome of *Brassica napus* using a label-free shotgun proteomic approach to elucidate plant response mechanisms to Cd toxicity.

**Results:**

We identified and quantified 672 proteins; 67% were predicted to be secretory, and 11% (73 proteins) were unique to Cd-treated samples. Cd stress caused statistically significant and biologically relevant abundance changes in 28 xylem sap proteins. Among these proteins, the metabolic pathways that were most affected were related to cell wall modifications, stress/oxidoreductases, and lipid and protein metabolism. We functionally validated a plant defensin-like protein, BnPDFL, which belongs to the stress/oxidoreductase category, that was unique to the Cd-treated samples and played a positive role in Cd tolerance. Subcellular localization analysis revealed that BnPDFL is cell wall-localized. In vitro Cd-binding assays revealed that BnPDFL has Cd-chelating activity. *BnPDFL* heterologous overexpression significantly enhanced Cd tolerance in *E. coli* and *Arabidopsis*. Functional disruption of *Arabidopsis* plant defensin genes *AtPDF2.3* and *AtPDF2.2*, which are mainly expressed in root vascular bundles, significantly decreased Cd tolerance.

**Conclusions:**

Several xylem sap proteins in *Brassica napus* are differentially induced in response to Cd treatment, and plant defensin plays a positive role in Cd tolerance.

**Electronic supplementary material:**

The online version of this article (10.1186/s12870-019-1895-7) contains supplementary material, which is available to authorized users.

## Background

Cadmium (Cd) is a toxic non-essential element for plants. Plant vascular systems consist of xylem and phloem; their long-distance transport of various compounds allows plants to adapt to different environments [1–7]. The accumulation of Cd in a plant shoot is mainly determined by the plant’s capacity to transport xylem sap long distances. Xylem sap is comprised of proteins, plant hormones, ions, and other molecules [7–12]. Xylem sap proteomic research can be used to illustrate the impact of long-distance transport mechanisms in plants in response to Cd stress.

The long-distance transport of xylem sap is mainly driven by transpiration and root pressure [13]. Proteomic studies of xylem sap in many plant species have been reported over the past three decades [14–26]. Of the identified proteins, most are categorized as classical secretion proteins, and function annotation has revealed that they play important roles in plant growth, development, and stress responses [14–26]. With regards to stress responses, in one study, the xylem sap proteome changed in response to iron and manganese deficiencies in tomato (*Solanum lycopersicum*) [25], and in another, nitrogen under- and over-supply induced distinct protein responses in the xylem sap of maize [26].

Long-distance signaling allows plants to adapt to and survive severe environmental stress conditions. To adapt to drought stress, the root-derived CLE25 is secreted by the procambium of the xylem, transported from the roots to leaves where it is recognized by BAM1 and BAM3, then it upregulates the ABA biosynthesis gene, *NCED3* and increases ABA accumulation, which in turn promotes stomatal closure in *Arabidopsis* [27]. Studies of the relay of C-TERMINALLY ENCODED PEPTIDE 1 (CEP1), CEP RECEPTOR 1 and 2 (CEPR1 and CEPR2), and CEP DOWNSTREAM (CEPD) have revealed that multiple layers of integration between local and systemic signals in roots and shoots orchestrate both the soil microenvironment and internal demand, thereby stimulating nitrate acquisition in nitrate-rich patches [28, 29]. However, it remains largely unknown how proteins change in response to Cd in xylem sap.

Proteomic change and long-distance transport from roots to shoots in xylem sap is an important mechanism for adapting to environmental stress [27–29]. Here, we investigate how *Brassica napus* plants respond to Cd via comprehensive analysis of proteomic changes in *Brassica napus* xylem sap, with the aim of elucidating plant response mechanisms to toxic Cd stresses. This study identifies many differentially-induced proteins in response to Cd treatment in the xylem sap of *Brassica napus* and functionally validates the hypothesis that plant defensins positively regulate Cd tolerance.

## Results

### Cadmium treatment affected *Brassica napus* seedling growth and physiological traits

**Fig. 1:**
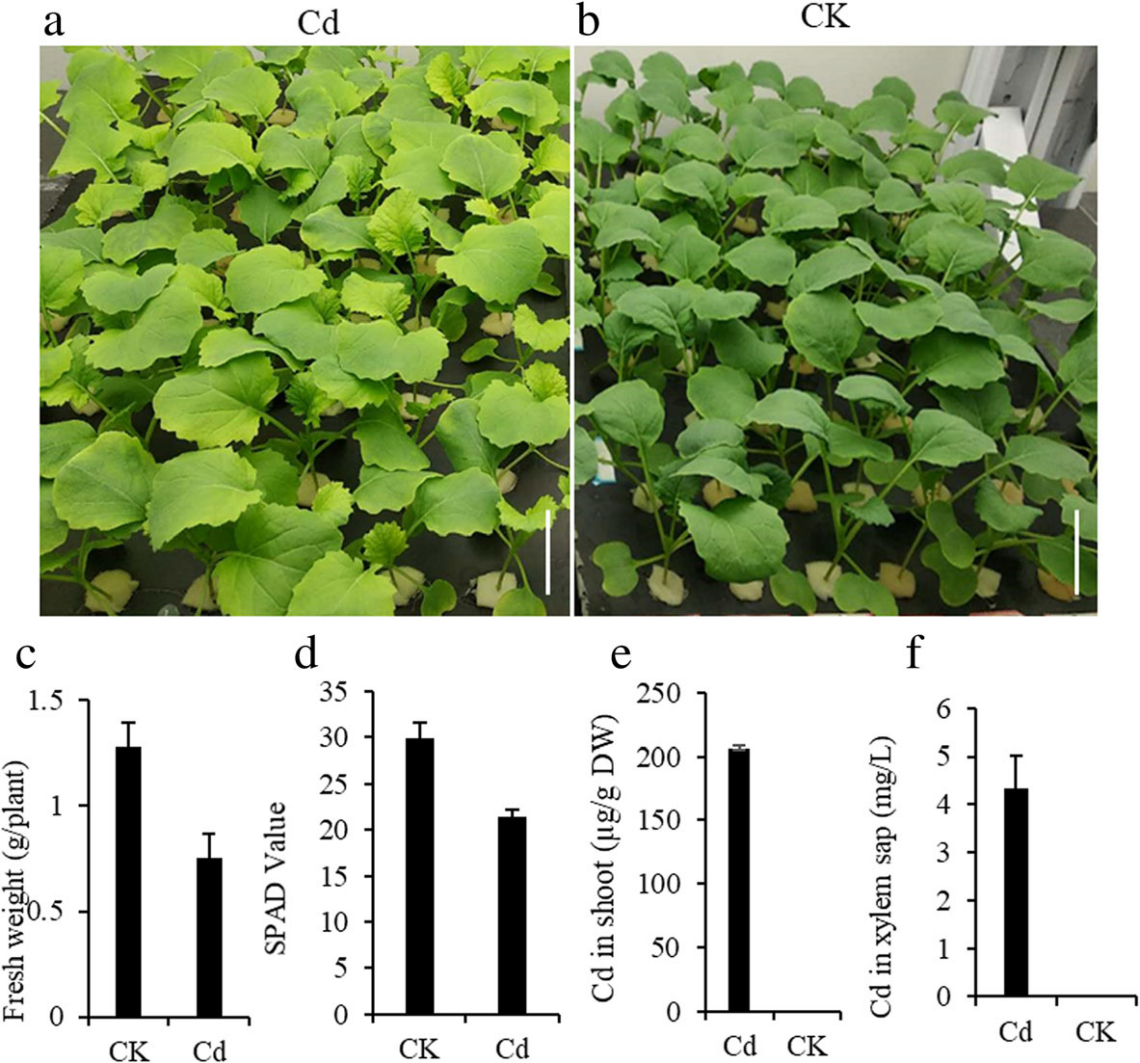
Physiological effects of Cd-addition to hydroponic *Brassica napus* plants. *Brassica. napus* cultivar ‘Xiang-you 15’ (‘XY15’) seedlings were hydroponically cultivated. At 2 weeks of age, the hydroponically grown *Brassica napus* seedlings were exposed to **a** 10 and **b** 0 μM Cd. After three days, **c** fresh weight, **d** SPAD value and Cd in **e** shoots and **f** xylem sap were determined. Scale bars, 5 cm. Data are mean ± SD, *n* = 5

*Brassica napus* plants (2 weeks-old), hydroponically grown with 10 μM Cd, showed serious symptoms 3 days after treatment. At sampling time (Day 17), a marked chlorosis was observed on younger leaves (Fig. 1a, b). The average leaf chlorophyll concertation in these plants (21.4) was significantly lower than that measured in the control plants (29.9) (Fig. 1c). Plant fresh weight significantly decreased after Cd treatment (Fig. 1d). Accordingly, the concentrations of Cd determined by ICP-MS in the shoot and xylem sap of Cd treated plants were 206 μg/g dry weight and 4.3 mg/L respectively (Fig. 1e and f).

### *Brassica napus* xylem sap proteins detected by Coomassie blue staining

**Fig. 2:**
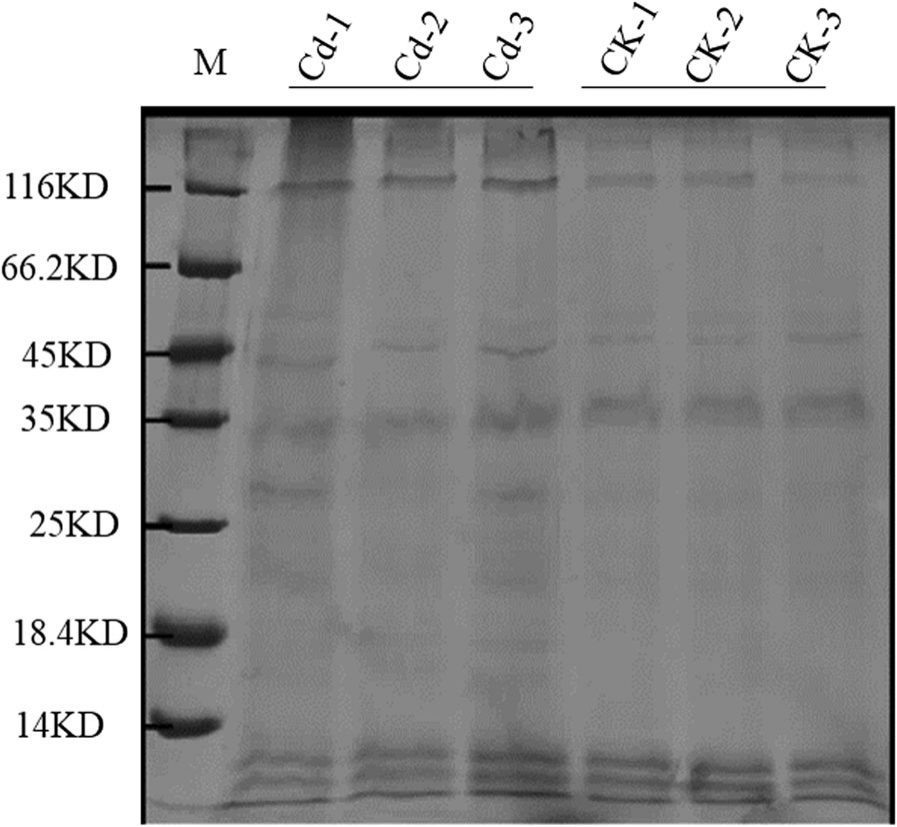
Separation of xylem sap proteins by SDS-PAGE. Proteins from the xylem sap were concentrated then separated by SDS-PAGE. The gel was stained with Coomassie brilliant blue. M are protein molecular mass markers (kDa). CK-1, CK-2, CK-3, Cd-1, Cd-2, and Cd-3 represent biological replicates xylem sap samples from control and Cd-treated *Brassica napus* plants respectively

We obtained 15–20 ng/μl protein from *Brassica napus* xylem sap using the root pressure method. SDS–PAGE analysis of xylem sap (15 μl) followed by Coomassie blue staining revealed different protein compositions in these saps (Fig. 2). The protein molecular weights ranged from approximately 14 to 116 kd. Cd significantly increased protein abundance at 116 kd (Fig. 2). To identify proteins in these samples that are below the limit of detection by Coomassie blue staining, we utilized the ‘shotgun analysis’ proteomic method.

### Identification of xylem sap proteins

In-solution digestion was carried out to determine the protein contents of xylem sap samples from control and Cd-treated *Brassica napus* plants (CK-1, CK-2, CK-3, and Cd-1, Cd-2, and Cd-3, respectively) (Additional file 1). The LC-MS/MS analysis detected 672 proteins in the *Brassica napus* xylem sap, 460 proteins in the CK samples, and 468 in the Cd-treated samples (Table 1). Of the 672 proteins, 244 were reliably identified and quantified with at least two peptides.

**Table 1:**
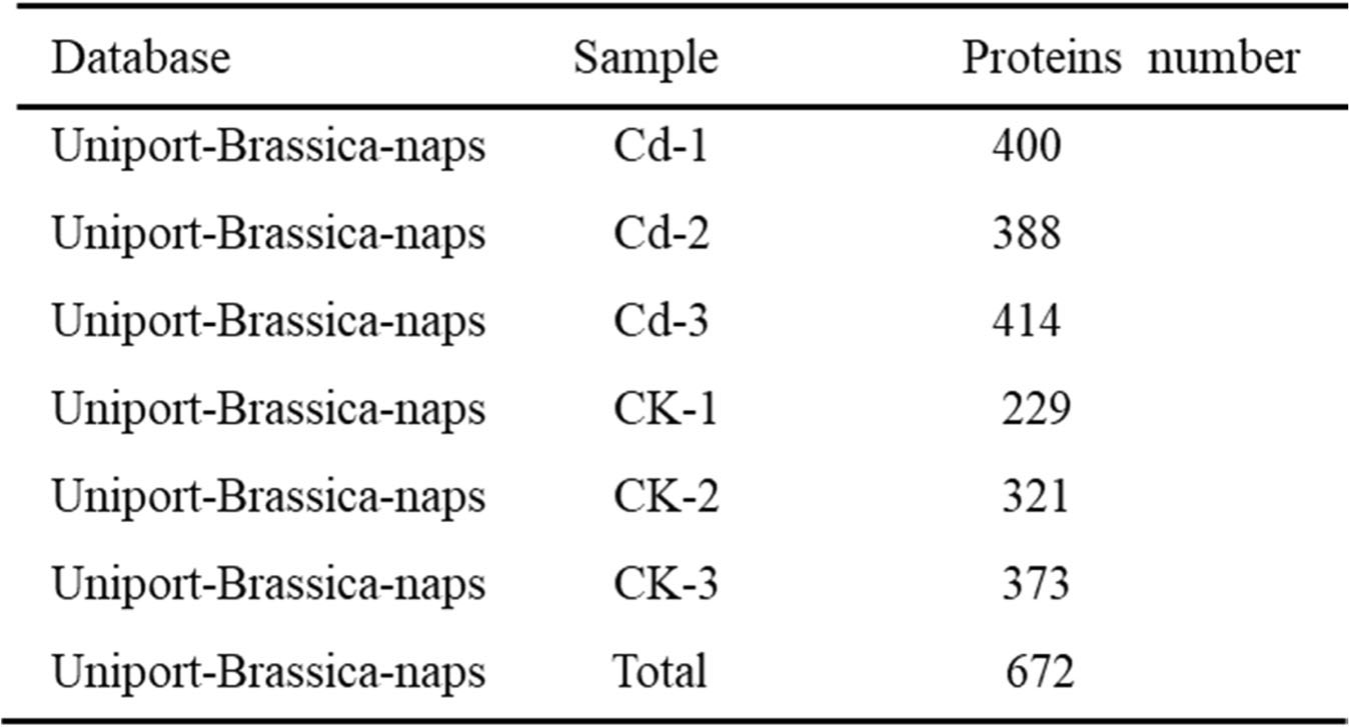
Proteins identified using label-free shotgun analyses in the xylem sap of *Brassica napus*

Bioinformatics analysis with the web tool TargetP and SecretomeP among the identified proteins revealed that 450 of the 672 identified and quantified proteins (67%) belong to the secretory pathway. Among these secretory proteins, 282 (42% of the total identified in the xylem sap) were classified as classical secretory proteins (CS), those containing secretion signal peptides, whereas 168 (25%) were classified as non-classical secretory proteins (NCS). The remaining 222 proteins (33%) were predicted to be non-secretory proteins (NS) (Additional file 2).

Of the proteins identified in at least two independent samples, 268 were found in both control and Cd-treated samples, 50 were unique to control samples, and 73 were unique to Cd-treated samples (Additional file 3a). For the xylem sap proteins of the control plants, shotgun label free LC-MS/MS proteomic analysis identified 229, 321 and 373 plant proteins from CK-1, CK-2 and CK-3, respectively, and 400, 388 and 414 plant proteins from Cd-1, Cd-2 and Cd-3, respectively (Table 1). Among these, 145 proteins were common among the control plants (63.3% of CK-1, 45.2% of CK-2 and 38.9% of CK-3), and 331 proteins were common among the Cd-treated plants (82.8% of Cd-1, 85.3% of Cd-2, and 80.0% of Cd-3) (Additional file 3 b, c). Differences in the number of proteins identified in the xylem sap samples between the control and Cd-treated samples could be due to Cd stress.

The molecular masses of the *Brassica napus* xylem sap plant proteins ranged from 1.5 kDa (A0A078JEE5, Uncharacterized protein) to 228.4 kDa (A0A078F4P9, Uncharacterized protein). However, the majority of plant proteins (90%) had molecular masses between 5 and 50 kDa (Additional file 4 a), which coincided with the band patterns observed in the one-dimensional SDS-PAGE (Fig. 2). These results suggest that *Brassica napus* xylem sap proteins mainly consist of relatively small sized proteins. The average andromeda score for *Brassica napus* xylem sap plant proteins was 89.55 and most of the identified proteins (88%) had an andromeda score greater than 60 (Additional file 4 b). The peptide coverage distribution of *Brassica napus* xylem sap plant proteins ranged from 1 to 90%, while the identified peptide length distribution of *Brassica napus* xylem sap plant proteins ranged from 7 to 33 amino acid residues (Additional file 4 c, d).

### Effect of cd treatment on the xylem sap proteome

**Fig. 3:**
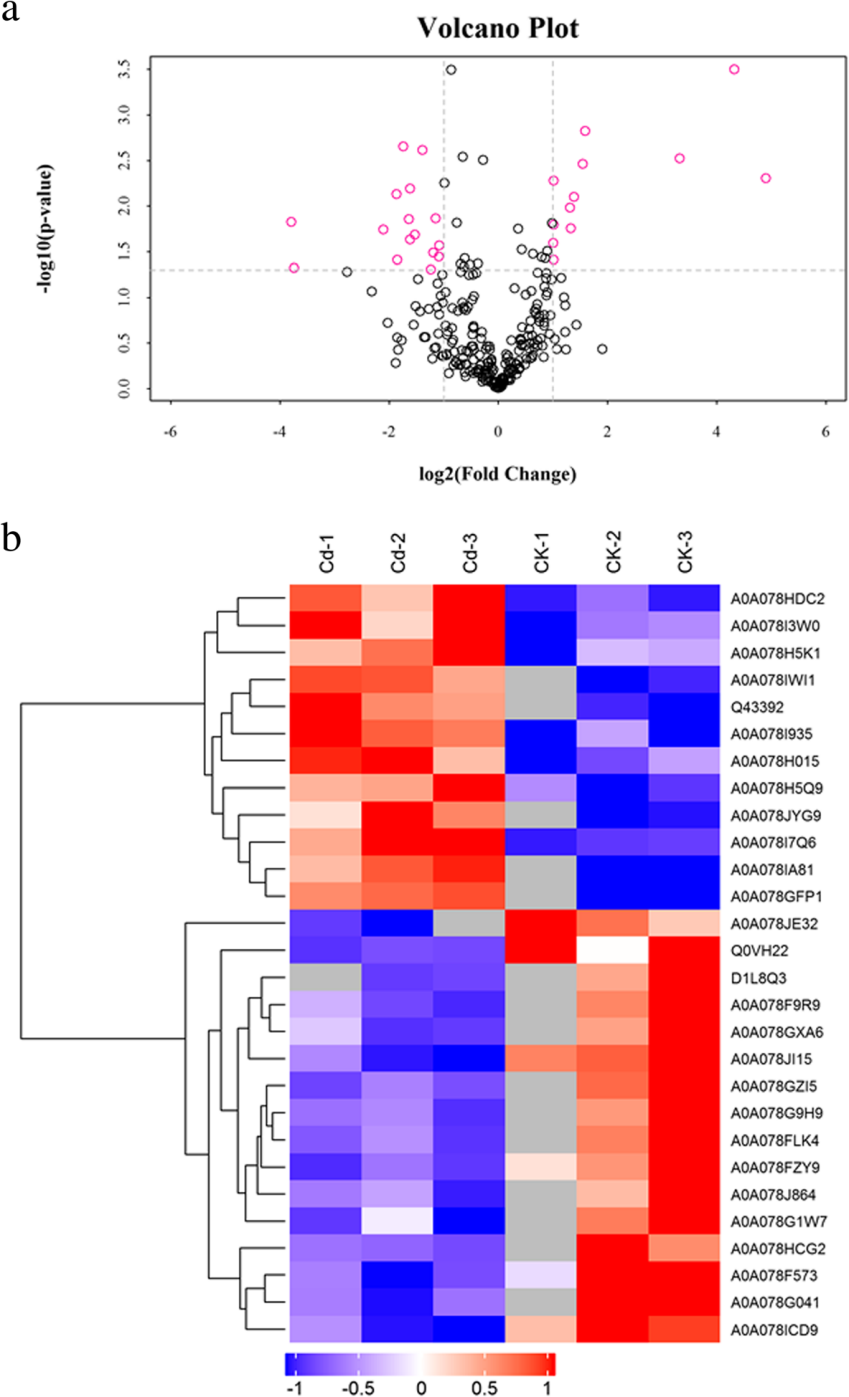
Effect of Cd on the xylem protein profile as revealed by label-free shotgun analyses. At two weeks of age, the hydroponically grown *Brassica napus* seedlings were exposed to 0 and 10 μM of Cd for three days. **a** Volcano scatter plot showing the identified and quantified proteins in xylem sap. Proteins unaffected by Cd are depicted in black, and proteins changing as a result of Cd (ANOVA, *p* ≤ 0.05) are depicted in red (left represents decreasing, right represents increasing). **b** Cluster of proteins controlled by Cd (ANOVA *p* < 0.05). Heatmaps report high (red) and low level (blue). CK-1, CK-2, CK-3, Cd-1, Cd-2, and Cd-3 represent biological replicates xylem sap samples from control and Cd-treated *Brassica napus* plants respectively

Cd treatment caused statistically significant (ANOVA, *p* ≤ 0.05) and biologically relevant (fold ≥2 or fold ≤0.5) changes in 28 proteins (Fig. 3). Among them, 12 proteins showed significant increases (fold change ≥2 in Table 2) and of these, 9 were classified as secretory (8 CS and 1 NCS). The remaining three proteins were classified as non-secretory proteins (Table 2). Based on the functional classification of the 12 proteins that increased in abundance, stress/oxido-reductases and protein synthesis metabolism were the most represented categories (Table 2). Remarkable increases in abundance (greater than 20-fold) were observed in two proteins: one cysteine-rich secretory protein, Antigen 5, that belongs to the pathogenesis-related 1 protein superfamily protein (A0A078IA81) and one osmotin-like protein (A0A078GFP1). These data will be useful for identifying candidate genes for further study.

**Table 2:**
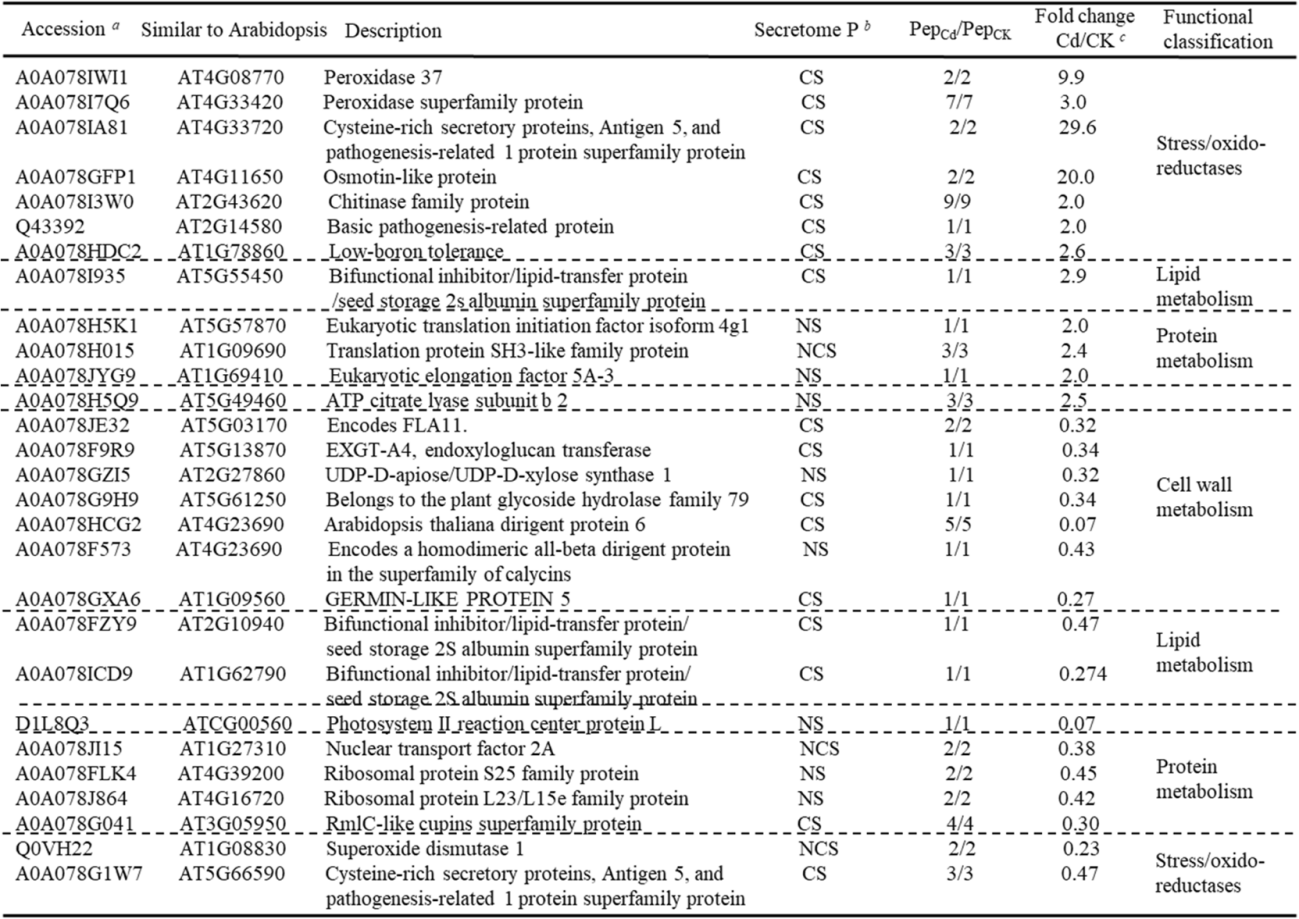
Proteins that significantly changed in abundance among the identified proteins affected by Cd (ANOVA, *p* ≤ 0.05 and fold ≥2 or ≤ 0.5)

^*a*^Accession indicates protein UniProt database entry

^*b*^The SecretomeP column indicates results from subcellular classification. CS = Classical secretory proteins, NCS = non-classical secretory proteins, NS = non-secretory proteins

Pep_Cd_/Pep_CK_ indicates the number of peptides assigned to a protein and number of peptides used for quantification

^*c*^Abundance changes (Fold Cd/control) were calculated by dividing the relative mean abundances in mean Cd by that of the control samples (mean control)

Cd treatment caused relative decreases in the abundance of 16 proteins (fold change < 0.5 in Table 2), and from these 11 were classified as secretory proteins (9 CS and 2 NCS). The remaining 5 proteins were classified as non-secretory proteins. Functional classification of the 16 proteins that decreased in abundance showed that cell wall modification, lipid metabolism and protein metabolism were the most represented categories (Table 2). Most increases were moderate, ranging between 2- and 3-fold, but remarkable decreases in abundance (higher than 14-fold) were observed in two proteins: a photosystem II reaction center protein L(D1L8Q3) and an *Arabidopsis thaliana* dirigent protein 6-like protein (A0A078HCG2).

Among the 672 proteins identified in the xylem sap, 73 proteins were unique to the Cd-treated plants and from these, 46 were classified as secretory (26 CS and 20 NCS). The remaining 27 proteins were classified as non-secretory proteins. Functional classification of the 46 secretory proteins revealed that cell wall metabolism, stress/oxido-reductase, protein synthesis/degradation and carbohydrate metabolism proteins were the most represented categories (Table 3). These proteins may specifically regulate Cd stress responses.

**Table 3:**
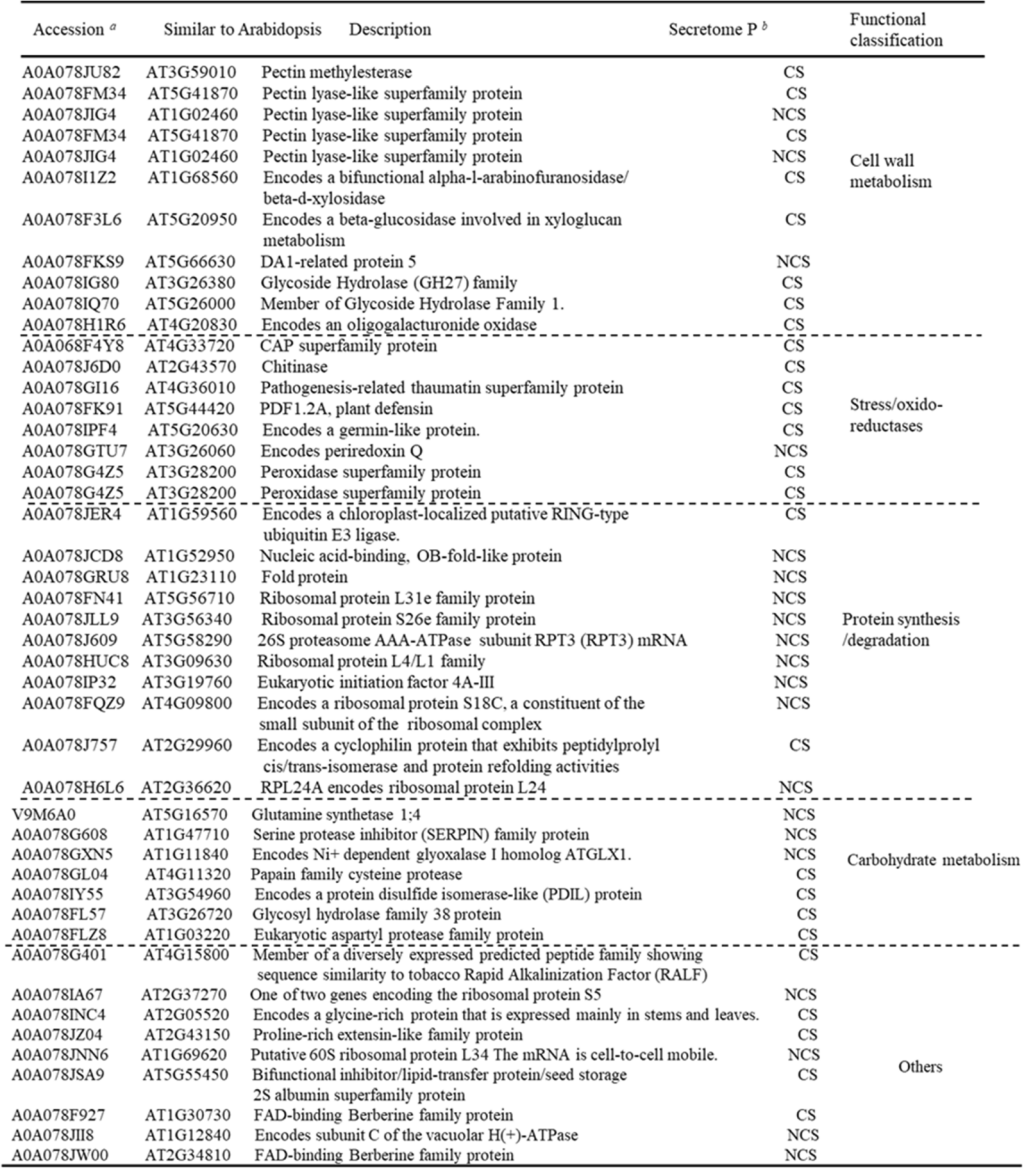
List of identified proteins unique to Cd-treated xylem sap samples from *Brassica napus*

^*a*^ Accession indicates protein UniProt database entry

^*b*^ The SecretomeP column indicates results from subcellular classification. CS = Classical secretory proteins, NCS = non-classical secretory proteins

### Defensin-like proteins in *Brassica napus* xylem sap

**Fig. 4:**
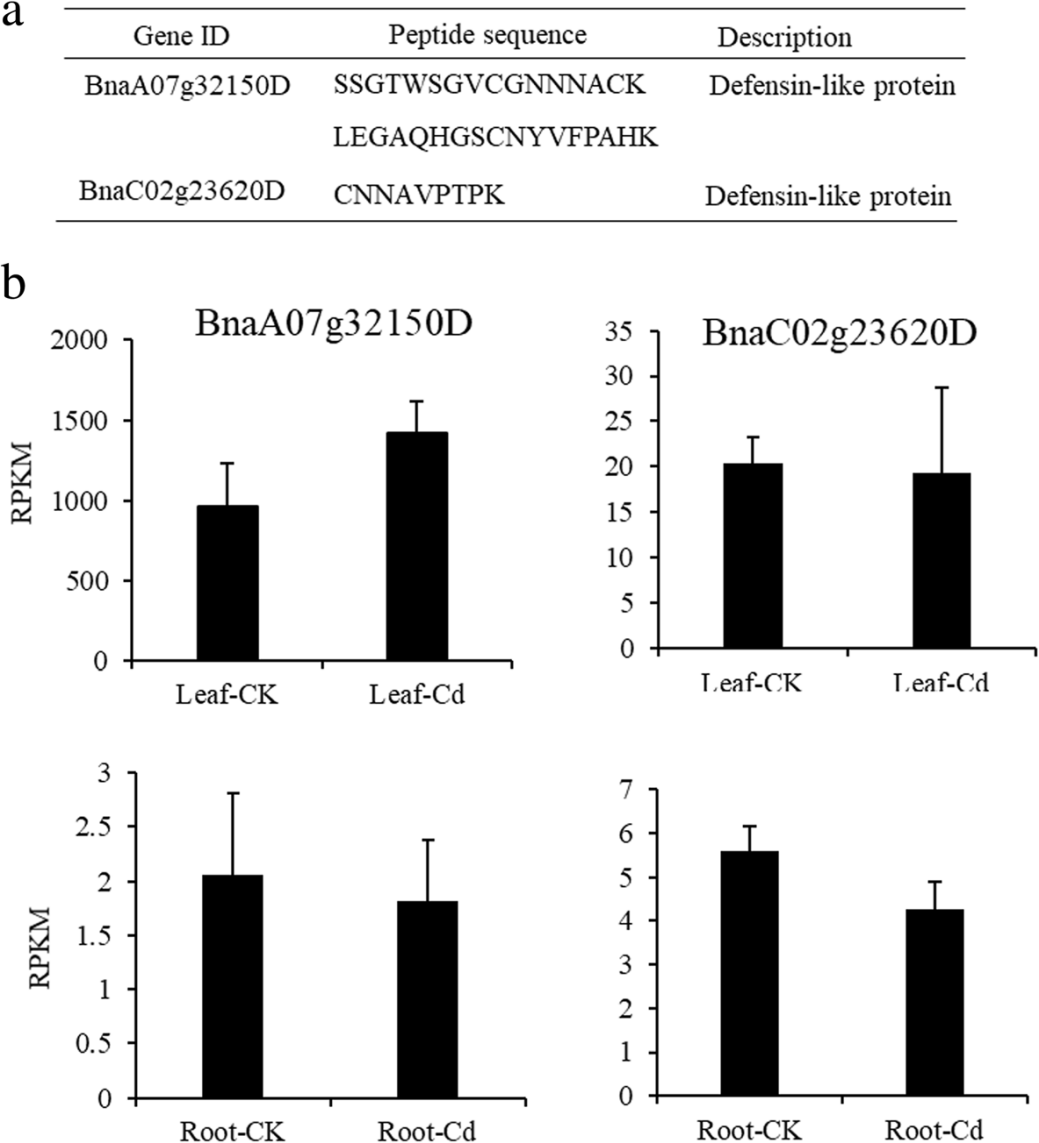
Defensin-like proteins identified in in the xylem sap of *Brassica napus* and expression. **a** Two defensin-like proteins were identified in the xylem sap of *Brassica napus* by LC-MS/MS. **b** The expression levels of two defensin-like genes in *Brassica napus* leaves and roots based on RNA sequencing. At two weeks of age, the hydroponically grown *Brassica napus* seedlings were exposed to 0 and 10 μM of Cd for three days. Data are mean ± SD, *n* = 3

We identified two proteins in *Brassica napus* xylem sap that were similar to the rice defensin-like proteins CAL1 that play important roles in Cd efflux and allocation [30]. The related peptides that we identified could be clearly mapped to the products of two different genes of this family based on their amino acid sequences (Fig. 4a). We examined the expression patterns of genes for the defensin-like proteins detected in xylem sap using RNA sequences from roots and leaves of *Brassica napus* plants. The mRNAs for these proteins were distributed mainly in *Brassica napus* leaves (Fig. 4b). The relative expression of *BnaA07g32150D* and *BnaC02g23620D* in leaves and roots were unaffected by Cd treatment. We used *BnaC02g23620D,* named *BnFDEL* and uniquely present in the Cd-treated xylem sap, for further functional validation.

### Functional identification of *BnPDFL* in vitro

**Fig. 5:**
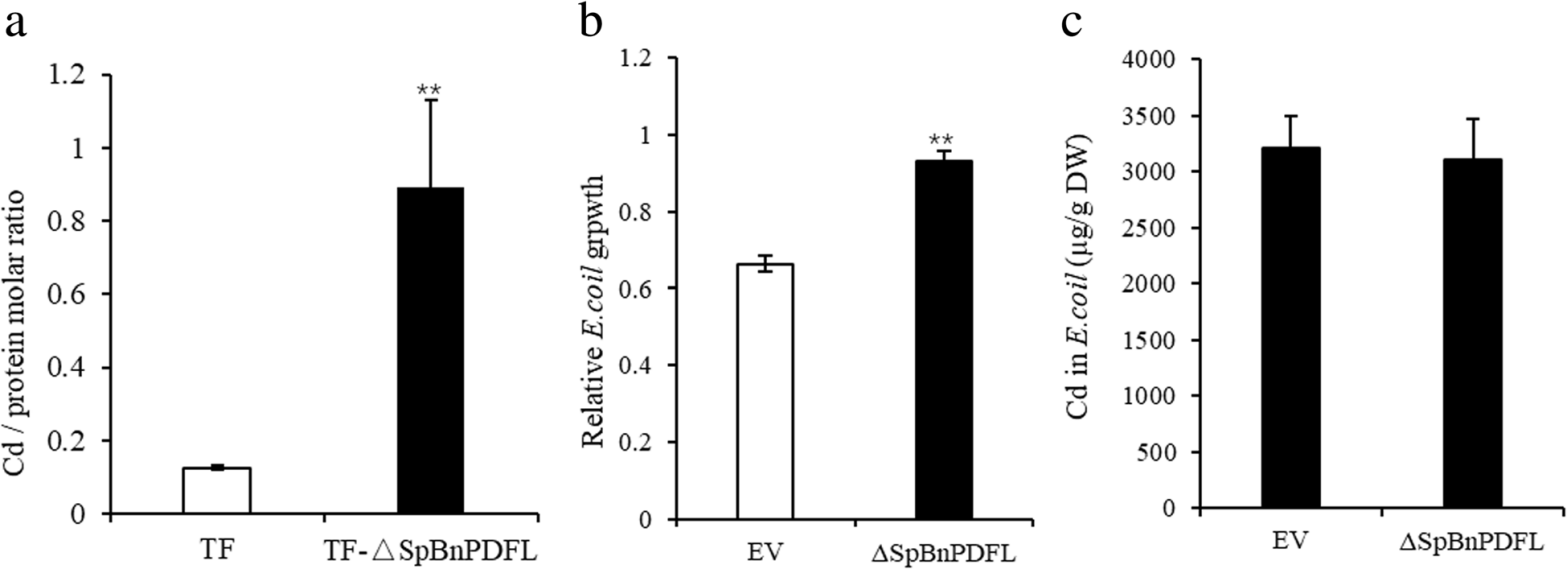
In vitro Cd binding, tolerance and accumulation assay of ΔSpBnPDFL in *E. coli.* **a** In vitro Cd binding assay. TF represents the *E. coli* trigger factor protein that fused to the N-terminus of target proteins, and the secretion signal peptide (SP)-deleted *BnPDFL* (TF-ΔSpBnPDFL) were used to transform *E. coli*. The molar ratio of Cd against TF-ΔSpBnPDFL protein purified from *E. coli* cells grown with 100 μM CdCl_2_ for 10 h was determined by ICP-MS. **b** Heterologous overexpression of *ΔSpBnPDFL* enhanced Cd tolerance in *E. coli.* Relative growth rates for *E. coli* strains expressing empty vector or ΔSpBnPDFL supplemented with 0, 200 μM CdCl_2_ for 6 h. **c** Cd concentration in *E. coli* strains from (**b**) was determined by ICP-MS. Data are mean ± SD, *n* = 4. Significant differences were determined by Student’s t-test (**P < 0.01)

Bioinformatics analysis showed that *BnPDFL* encodes a defensin family protein of 85 amino acids, consisting of a cysteine-rich domain and a secretion signal peptide. The probable signal peptide cleavage site is between position 25 and 26 amino acids (Additional file 5 a). The amino acid sequences BnPDFL are not similar to those of PDF proteins from other plants (Additional file 5 b). Metal binding assays were performed using purified fusion protein from *E. coli* transformants treated with 100 μM CdCl_2_ as described [31]. An ΔSpBnPDFL/Cd molar ratio of ~ 1 was detected for the ΔSpBnPDFL fusion protein (Fig. 5a). Heterologous overexpression of *ΔSpBnPDFL* enhanced Cd tolerance in *E. coli* (Fig. 5b), while it had no effect on Cd accumulation (Fig. 5c), consistent with the model indicating that BnPDFL mediated the chelation of Cd to enhance Cd tolerance.

### Heterologous overexpression of *BnPDFL* enhanced cd tolerance in *Arabidopsis*

**Fig. 6:**
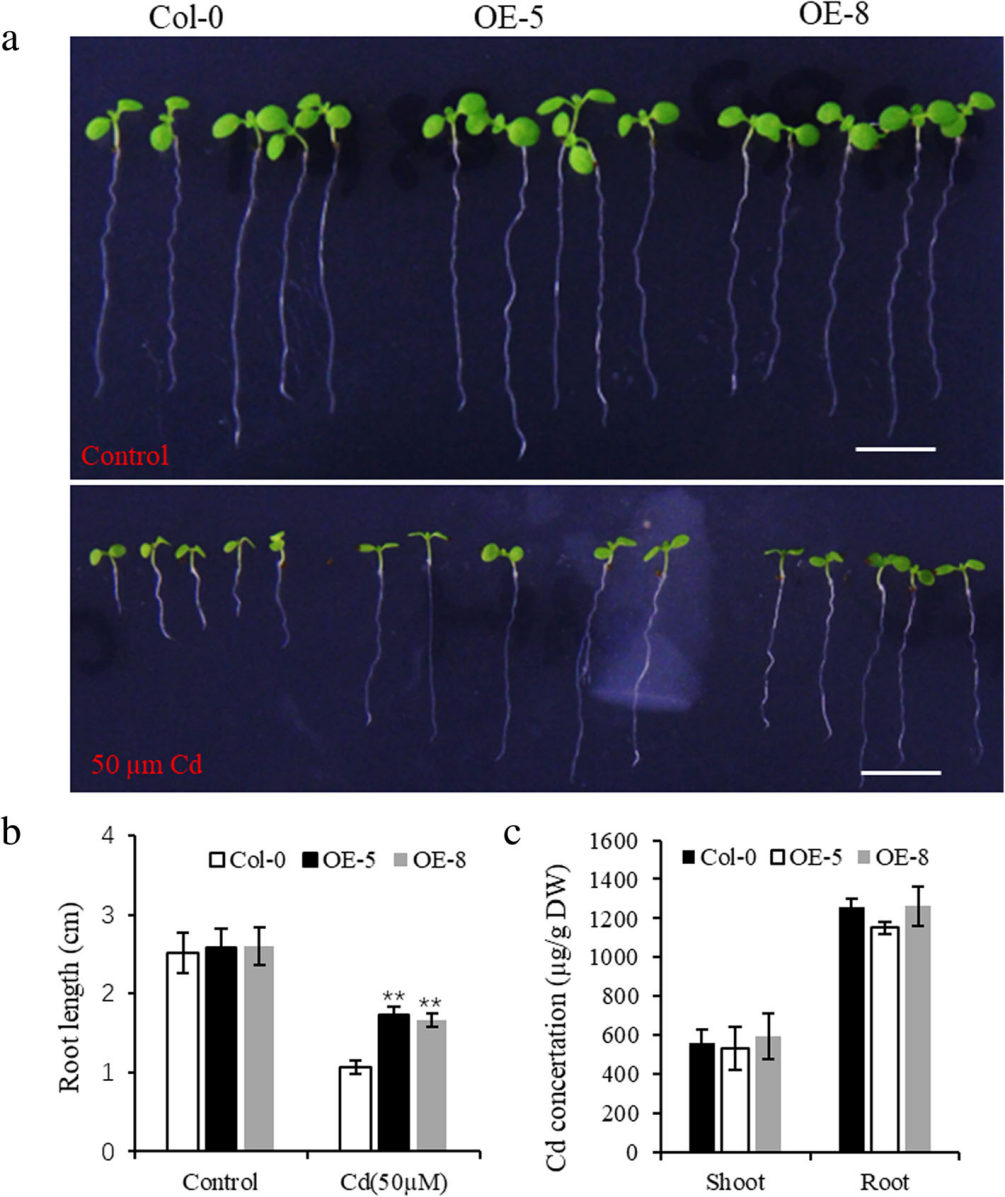
Heterologous overexpression of *BnPDFL* enhanced Cd tolerance in *Arabidopsis.* **a** Surface sterile seeds plated onto half-strength MS medium supplemented with 0 μM and 50 μM CdCl2 and grown vertically for 1 week. OE-5 and OE-8 are independent lines transgenic for 35S:: *BnPDFL -mRFP* in wild-type Col-0 background. Scale bars, 1 cm. **b** Root elongation was determined in plants grown under conditions in A. **c** Four weeks old plants grown in hydroponics were exposed to 10 μM Cd for 3 days. Cd concertation in *Arabidopsis* shoots and roots. Data are mean ± SD, *n* = 15 in **b**, or 5 in **c**. Significant differences were determined by Student’s t-test (**P < 0.01)

Our previous study suggested that defensin-like gene mediates cadmium tolerance or accumulation [30–32]. To examined the effect of heterologous overexpressing *BnPDFL* on metal tolerance in *Arabidopsis*, we transformed Arabidopsis with construct BnPDFL-mRFP driven by the 35S promoter. Subcellular localization assay revealed that BnPDFL is a cell wall localized protein (Additional file 6). When germinated without heavy metals, the growth of overexpression lines OE-5 and OE-8 was similar to that of the wild-type Col-0 (Fig. 6a). However, when germinated on 50 μM CdCl_2_, lines OE-5 and OE-8 grew longer roots than did the wild-type control (Fig. 6b). However, no significant differences were observed in shoot and root Cd levels between wild-type Col-0 and overexpression lines (Fig. 6c). These data indicated that heterologous overexpression of *BnPDFL* enhanced Cd tolerance in *Arabidopsis*.

### Functional disruption of plant defensin decreased cd tolerance in *Arabidopsis*

**Fig. 7:**
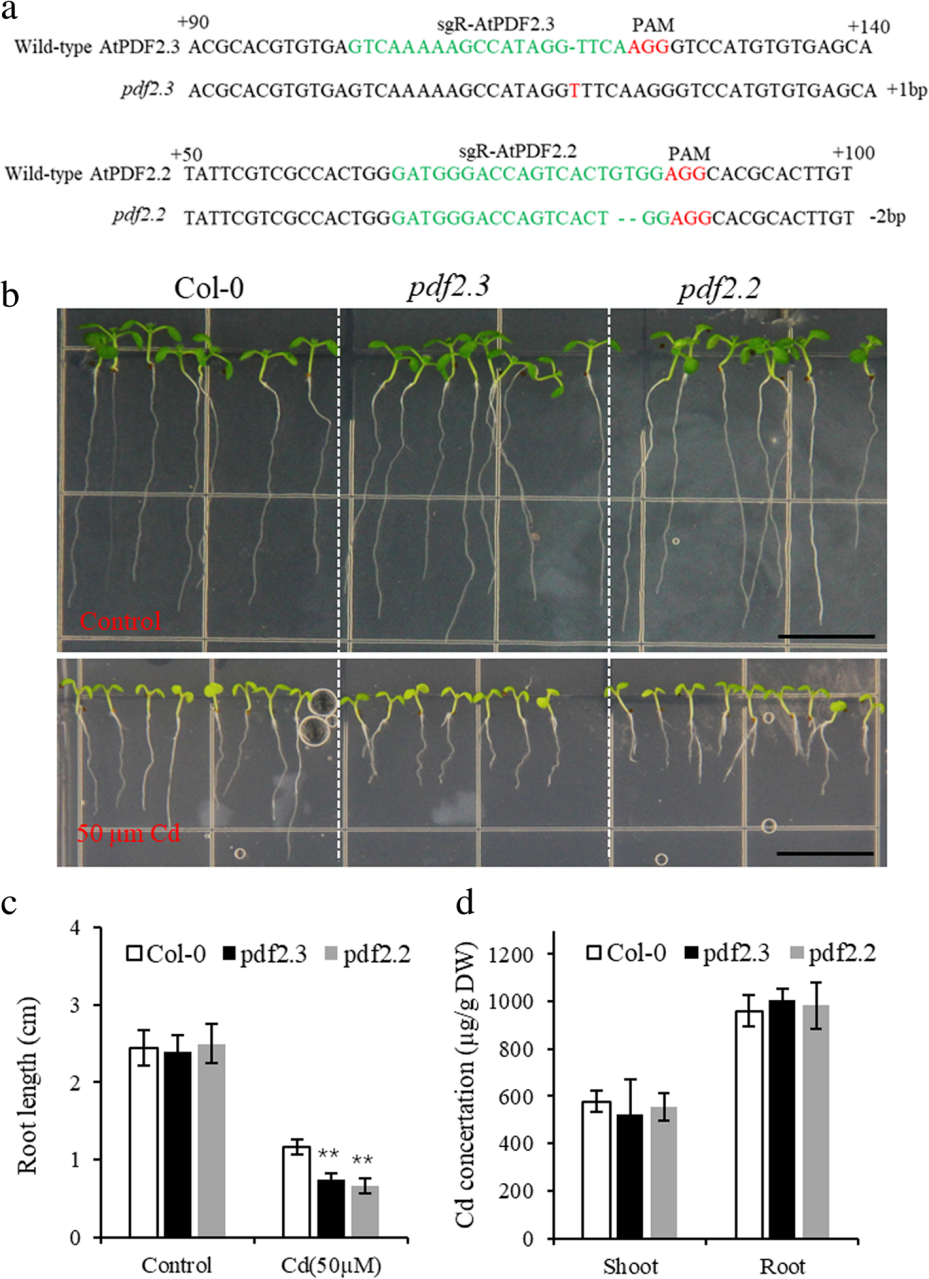
Loss of function of plant defensin decreased Cd tolerance in *Arabidopsis.* **a** Identification of plant defensin mutants generated by CRISPR/Cas9. *pdf2.3* represent mutants with single T insertion in plant defensin gene *AtPDF2.3* coding region, *pdf2.2* represent mutants with two base GT deletions in plant defensin gene *AtPDF2.2* coding region, with Col-0 background. **b** Col-0, *pdf2.3* and *pdf2.2* seedlings grown in half-strength MS medium in the absence (top) or presence (bottom) of Cd for one week. Scale bars, 1 cm. **c** Root elongation was determined in plants grown under conditions in **b**. **d** Four weeks old plants grown in hydroponics were exposed to 10 μM Cd for 3 days. Cd concertation in *Arabidopsis* shoots and roots. Data are mean ± SD, n = 15 in **c**, or 5 in **d**. Significant differences were determined by Student’s t-test (**P < 0.01)

To further show that plant defensin plays a positive role in Cd tolerance, we generated two homozygous knockout mutants of plant defensin *AtPDF2.2* and *AtPDF2.3* using clustered regularly interspaced short palindromic repeats/associated protein 9 (CRISPR/Cas9) technology (Fig. 7a). To clarify the expression pattern of AtPDF2.2 and AtPDF2.3, we generated AtPDF2.2 and AtPDF2.3 promoter-driven GUS transgenic *Arabidopsis*. AtPDF2.3 promoter-driven GUS signals were detected in root and shoot vascular bundles (Additional file 7 a, b), while AtPDF2.2 promoter-driven GUS signals were detected mainly in root vascular bundles, root hairs and cotyledons (Additional file 7 c, d). The seedling metal tolerance assay result obviously showed that the roots of the *pdf2.3* and *pdf2.2* mutants were more sensitive to Cd compared with wild-type Col-0 (Fig. 7b, c). While no significant differences were detected in shoot and root Cd concertation between wild-type Col-0, *pdf2.3* and *pdf2.2* mutants (Fig. 7d). These results further confirmed that plant defensin conveyed Cd tolerance.

## Discussion

The proteomic approach used in this study identified and quantified 672 proteins in the xylem sap of *Brassica napus*, which is considerably higher than the 69 proteins previously identified in 2-DE [6], though a recent study found 643 proteins in tomato xylem sap using a similar shotgun method [25], indicating that the shotgun label-free analysis used in this study is very sensitive. Our results showed that nearly 67% of the identified xylem proteins were classified as secretory and 33% were identified as non-secretory, which could in part be due to cytoplasmic contamination. The relatively high percentage of non-secretory proteins observed here may also be due to the high sensitivity of the LC-MS/MS approach utilized, and their secretory mechanisms in the xylem sap proteome require further studies.

The plant cell has evolved many mechanisms to defend itself against Cd toxicity. Cell walls represent the most important physical and chemical barriers to prevent Cd from entering and damaging the protoplast [33]. The plant cell wall consists of primary and secondary cell walls and both have an array of detoxification mechanisms to cope with Cd stress. Pectin is the major component of primary cell walls, containing most of the negative charges, and it can sequester Cd. In the secondary cell wall, lignification can prevent Cd from entering and damaging the cell [33, 34]. The molecular mechanisms of cell wall responses to Cd stresses remain unknown. Here, we identified several proteins involved in cell wall metabolism that significantly changed in the xylem sap of Cd-treated plants: A0A078JE32, A0A078F9R9, A0A078GZI5, A0A078G9H9, A0A078HCG2, A0A078F573 and A0A078GXA6 significantly decreased under Cd stress (Table 2), while the methyl pectin enzyme A0A078JU82 was identified only in the xylem sap of the Cd-treated plants (Table 3). Xylem vascular tissue and the cell wall are part of the apoplast, and xylem parenchyma cells and cell wall localized proteins may be transported through the apoplast pathway to the xylem sap like CAL1 [30]. Homologues of these proteins have been associated with cell metabolism that mediates abiotic stress response in *Arabidopsis* [34–38]. Our results indicated that the plant cell wall plays an important role in Cd detoxification and accumulation, and we identified many candidate genes for further study.

Cadmium can induce the production of plant reactive oxygen species that are harmful to plant cells via indirect mechanisms [39], and it is important to maintain cellular redox balance. Previous studies revealed that antigen 5 and pathogenesis-related 1 protein (CAP) superfamily negatively regulate salt-stress tolerance in *Arabidopsis* [40]. Here, the CAP/oxido-reductase related proteins A0A078IWI1, A0A078I7Q6, A0A078IA81, A0A078GFP1, A0A078I3W0, Q43392 and A0A078HDC2 significantly increased and Q0VH22 and A0A078G1W7 decreased under Cd stress in *Brassica napus* xylem sap (Table 2). This suggests that the antioxidant defense system attempted to restore a disturbed redox balance.

The long-distance root-to-shoot transport of phytochelatins mediate Cd tolerance and accumulation in *Arabidopsis* [41]. The defensin-like family of proteins are small basic cysteine rich peptides that inhibit the growth of a broad range of fungi [42]; however, the zinc/Cd-binding activity of human defensin 5 has been reported [43], plant defensin type 1 genes (*PDF1*s) enhance zinc tolerance [44, 45], and our previous study revealed that plant defensin-like gene, *CAL1*, AtPDF2.5 and AtPDF2.6 mediates cadmium tolerance or accumulation [30–32]. We identified two defensin-like genes *BnaA07g32150D* and *BnaC02g23620D* in the xylem sap of *Brassica napus* (Fig. 4). Functional analysis indicated that BnaC02g23620D (BnPDFL) had Cd-binding activity (Fig. 5a) and that heterologous overexpression of *BnPDFL* enhanced Cd tolerance in *E. coli* and *Arabidopsis* (Fig. 5b, c). Functional disruption of the plant defensins AtPDF2.2 and AtPDF2.2 significantly decreased Cd tolerance in *Arabidopsis* (Fig. 6b, c). Based on our results, plant defensin plays an important role in Cd tolerance via chelation. The BnaA07g32150D protein was still detected in xylem sap when no Cd was added, indicating that it might have other functions and may act as a long-distance hormone-like signal to mediate stress responses, like hepcidin in humans, CLE25 or CEP [27, 29, 46].

## Conclusions

In summary, we identified and quantified 672 proteins in the xylem sap of *Brassica napus*; 67% were predicted to be secretory, and 11% (73 proteins) were unique to the Cd-treated samples. Cd stress caused statistically significant and biologically relevant abundance changes in 28 xylem sap proteins. Among these proteins, the metabolic pathways that were most affected were cell wall modifications, stress/oxidoreductases, and lipid and protein metabolism. We functionally validated defensin-like protein *BnPDFL* in xylem sap, which acts as a Cd-binding peptide. Our study identified a number of xylem sap proteins from *Brassica napus* that are differentially induced in response to Cd treatments and confirmed that plant defensins positively regulate Cd tolerance.

## Methods

### Plant materials, xylem sap harvesting, and cd sensitivity analyses

*Brassica napus* cultivar ‘Xiang-you 15’ (‘XY15’) was provided from colleagues, which contains abundant xylem sap for proteomic analysis. *Arabidopsis* mutants (*pdf2.2* and *pdf2.3*) and transgenic lines were generated in our laboratory. XY15 seedlings were hydroponically cultivated according to the method described by Han et al. [47]. At 2 weeks of age, the hydroponically grown *Brassica napus* seedlings were exposed to 0 (control) and 10 μM Cd (treatment) for 3 days, then xylem sap samples were collected. Briefly, stems were cut with a razor blade 2–3 cm above the basal stems to collect xylem sap for 2 h. Xylem sap from the first 10 min was discarded to avoid contamination from damaged cells as described previously [25]. Xylem sap collected from 24 plants was pooled into one replicate, and a total of three replicates from 72 plants were used for each treatment. The xylem sap protein shotgun experiment was performed and analyzed by Shanghai Applied Protein Technology, Ltd. Leaf chlorophyll concentration was estimated using a SPAD 502 apparatus (Minolta Co., Osaka, Japan). The SPAD values of old expanded leaves were recorded at sampling time and an average per treatment was obtained. *Arabidopsis thaliana* plants were grown in quarter-strength hydroponic solution as described previously [48]. Seedling metal sensitivity assay were performed as described [32] with minor modification.

### Preparation of the protein samples for SDS-PAGE analysis and enzymolysis

The xylem sap samples were concentrated under vacuo and lyophilized. A buffer (200 μL SDT 4% SDS, 100 mM Tris/HCl, 0.1 M DTT) was added to the lyophilized samples, treated with ice bath sonication, centrifuged at 4 °C and 14,000×*g* for 15 min and then the supernatant was collected. The Bradford assay method was used to measure protein concentration. We used SDS-PAGE on 20 μL of the supernatant. Gels were stained with Coomassie Brilliant Blue. The filter aided proteome preparation method was used to carry out the trypsin enzymatic hydrolysis of each sample [49]. Samples were desalted using C18-SD Extraction Disk Cartridges, vacuum freeze-dried, dissolved in 0.1% formic acid, and quantified at OD280.

### Label free liquid chromatography-tandem mass spectrometry (LC-MS/MS)

An HPLC liquid phase system Easy nLC was used to separate the proteins. Buffer A contained 0.1% formic acid, and buffer B contained 0.1% formic acid acetonitrile (acetonitrile was 84%). The column was balanced with 95% buffer A. The samples were loaded into the Thermo Scientific EASY column (2 cm × 100 μM 5 μm-C18) using an automatic sampler, then separated in the analysis column Thermo scientific EASY column (75 μm × 100 mm 3 μm-C18) at a flow rate of 300 nl/min. The liquid phase gradient was as follows: 0–100 min, buffer B from 0 to 50%; 100–108 min, buffer B from 50 to 100%; 108–120 min, buffer B fluid maintained at 100%. The samples were detected using a Q-Exactive mass spectrometer (Thermo Finnigan) after separation from the capillary high-performance liquid chromatography. The detection parameters were as follows: analysis time, 120 min; detection methods, positive ions; mother ion scanning range, 300–1800 *m/z*; level of mass spectrum resolution, 70,000 at *m/z* 200; AGC (Automatic gain control) target, 1e6; level of maximum IT, 50 ms; number of scan ranges, 1; dynamic exclusion, 60.0 s. The peptide and the peptide fragment charge-mass ratios were determined according to the following methods: after every full scan, 10 pieces of the map (MS2 scan) were collected; MS2 Activation Type, HCD; isolation window, 2 *m/z*; resolution of the secondary mass spectrometry, 17,500 at *m/z* 200; microscans, 1; secondary maximum IT, 60 ms; normalized collision energy, 27 eV; Underfill ratio, 0.1%.

### Mass spectrometry data and bioinformatics analysis

The RAW file was retrieved from the client’s database (uniport-Brassica_napus_62788_2018-04-13) using the Proteome Discoverer 1.4 software. The search parameters were set as follows: missed cleavage was set to 2; static decoration was set to Carbamidomethy C; dynamic modification was set to Oxidation M; peptide tolerance was set to 20 ppm, ms/ms tolerance was set to 0.1 Da, peptide FDR was set to < 0.01, protein FDR was set to < 0.01. To assess the effect of Cd stress on the protein profile of tomato xylem sap, we calculated the ratio of normalized protein abundance in the Cd-treated and control samples. A volcano plot, showing the relationship between statistical significance [−log10(*p*-value)] and biological significance [log2(fold-change)], was used to describe the changes induced by Cd treatment on the xylem sap proteome. Only changes with a *p* ≤ 0.05 (ANOVA) and a normalized abundance ratio ≥ 2 or ≤ 0.5 were considered statistically significant and biologically relevant, respectively. Label-free quantification was used to determine protein relative content.

Go function annotation of the identified proteins was carried out using BLAST2GO software. This process can be summarized as sequence alignment, mapping, annotation, and annotation augmentation. The presence of signal peptides in proteins was assessed using TargetP (www.cbs.dtu.dk/services/TargetP), and SecretomeP (www.cbs.dtu.dk/services/SecretomeP) was used to assign proteins as classical secretory (CS) and non-classical secretory (NCS) [50–52].

### DNA constructs and transformation into *Arabidopsis*

A genomic fragment immediately upstream of the AtPDF2.2 (1385-bp) and AtPDF2.3 (962 bp) start codon were amplified with PCR using the primers ProAtPDF2.2 and ProAtPDF2.3, respectively (Additional file 8: Table S1). The resulting ProAtPDF2.2 and ProAtPDF2.3 promoter fragments were then sub-cloned into the binary vector pCAMBIA1300. To generate *pdf2.2* and *pdf2.3* mutants, the AtPDF2.2 and AtPDF2.3-specific guide RNA expression sequence was introduced into the CRISPR-Cas9 construct using primers sgR-AtPDF2.2 and sgR-AtPDF2.3, respectively (Additional file 8: Table S1), and the resulting 1300-bp fragment of Cas9 and AtPDF2.2 and AtPDF2.3-specific guide RNA expression cassettes were recovered by *Hind*III*/EcoR*I restriction digestion and sub-cloned into pCAMBIA1300 [53]. All the resulting constructs were transformed into *Arabidopsis* using the floral dip method [54]. Transgenic plants were screened using hygromycin B and confirmed by sequencing. To confirm the histological expression pattern of GUS driven by the proAtPDF2.2 and proAtPDF2.3 promoter, GUS histochemical staining was performed using a GUS histochemical assay kit (Real-Times, China) following the manufacturer’s protocol.

### Subcellular localization, protein purification and related assay

To determine the subcellular localization of BnPDFL in *Arabidopsis*, the 35S:: *mRFP* fragment was recovered from 35S:: *mRFP*/PA7 by *Hind*III*/Sac*I restriction digestion, and the resulting 35S:: mRFP fragment was inserted into pCAMBIA1300 to generate the construct 35S:: *mRFP*/pCAMBIA1300. The coding sequence of *BnPDFL* without a stop codon was PCR amplified using the primers OE-BnPDF (Additional file 8: Table S1). The resulting fragment was fused in-frame to the 5′ terminus of mRFP to generate the constructs 35S::*BnPDFL -mRFP*/pCAMBIA130 as described [31], and transformed into *Arabidopsis* using the floral dip method [54].

Fragment of ΔSpBnPDFL (a truncated form of BnPDFL representing the mature BnPDFL protein from amino acids 26 to 85) was PCR amplified using the primers TF-ΔSpBnPDFL (Additional file 8: Table S1) and cloned into the pCold-TF vector. Protein purification and related assay were performed as described previously [31]. The metal-to-protein stoichiometry was calculated according to the Cd and protein concentrations, as described previously [55]. ΔSpBnPDFL was also amplified by PCR using primers GST-ΔSpBnPDFL (Additional file 8: Table S1) and cloned into pGEX-2TK for metal sensitivity assays in *E. coli* as described previously [31].

### Statistical analysis

Data were analyzed using two-tailed Student’s *t* tests, and differences were deemed significant at *P* < 0.05 and extremely significant at *P* < 0.01.

### Accession numbers

Sequence data from this study can be found in the Arabidopsis Genome Initiative or GenBank/EMBL databases under the following accession numbers: *AtPDF2.1*(*At2g02120*)*, AtPDF2.2*(*At2g02100*)*, AtPDF2.3*(*At2g02130*)*, AtPDF2.4*(*At1g61070*)*, AtPDF2.5*(*At5g63660*)*,* and *AtPDF2.6*(*At2g02140*). *BnPDFL* (*BnaC02g23620D*) and *BnaA07g32150D* sequence can be found in uniport database.

## Additional files

Additional file 1: **Figure S1.** Simply Label free experiment design and analysis. (DOCX 370 kb)

Additional file 2: **Figure S2.** Subcellular localization prediction of the identified proteins. (DOCX 32 kb)

Additional file 3: **Figure S3.** Numbers of identified proteins in the control and Cd-treated samples. (DOCX 171 kb)

Additional file 4: **Figure S4.** Basic characteristics of proteins identified in *Brassica napus* xylem sap. (DOCX 391 kb)

Additional file 5: **Figure S5.** Bioinformatics analysis of BnPDFL. (DOCX 199 kb)

Additional file 6: **Figure S6.** BnPDFL is a Cell wall localized protein (DOCX 1838 kb)

Additional file 7: **Figure S7.** Histochemical localization of GUS activity in transgenic plants expressing the GUS reporter gene under the control of the proAtPDF2.3 or proAtPDF2.2 promoter. (DOCX 1753 kb)

Additional file 8: **Table S1.** Primers used in this study. (DOCX 104 kb)

## Data Availability

The datasets used and/or analyzed during the current study are available from the corresponding author on reasonable request.

## Abbreviations

1/2MS: one-half Murashige and Skoog
AGC: Automatic gain control
Cd: Cadmium
Cd-#: Cadmium-treated plant
CK-#: control plant
CS: Classical secretory proteins
LC- MS/MS: liquid chromatography-tandem mass spectrometry
NCS: non-classical secretory
NS: non-secretory
