# Supplementary figures and images for: Proteomic changes in the xylem sap of Brassica napus under cadmium stress and functional validation
Source: BMC Plant Biol. 2019 Jun 26;19:280. doi: 10.1186/s12870-019-1895-7 (PMC6595625; doi:10.1186/s12870-019-1895-7)

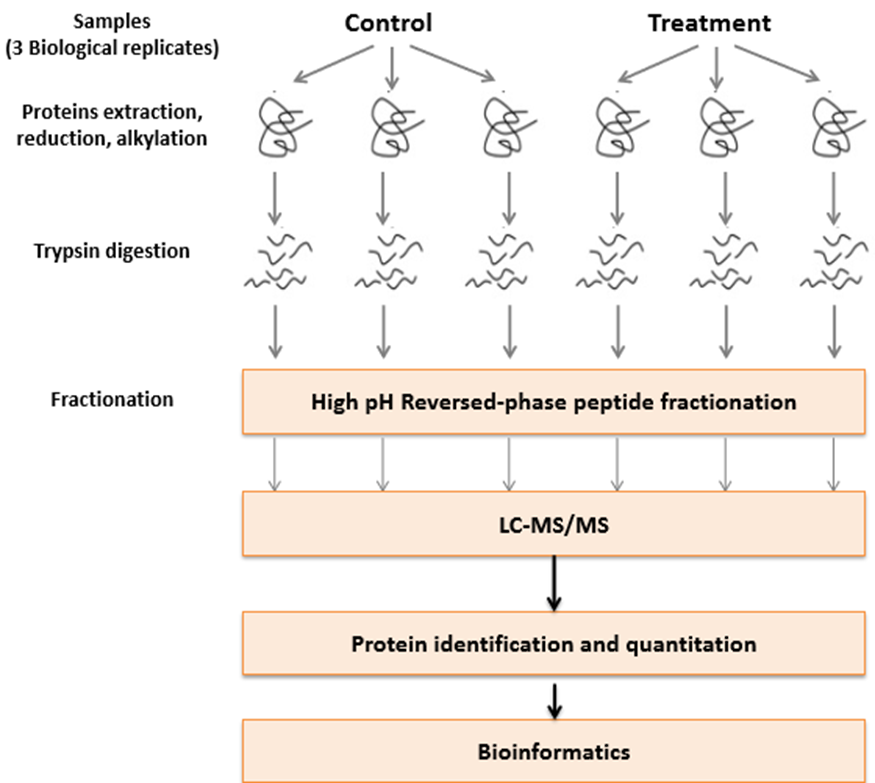


Additional file 1: **Figure S1**. Simply Label free experiment design and analysis.

Supplement: Supplementary file 1 — Figure S1. Simply Label free experiment design and analysis. (DOCX 370 kb) [file 12870_2019_1895_MOESM1_ESM.docx]

Additional file 8:Table S1*.* Primers used in this study


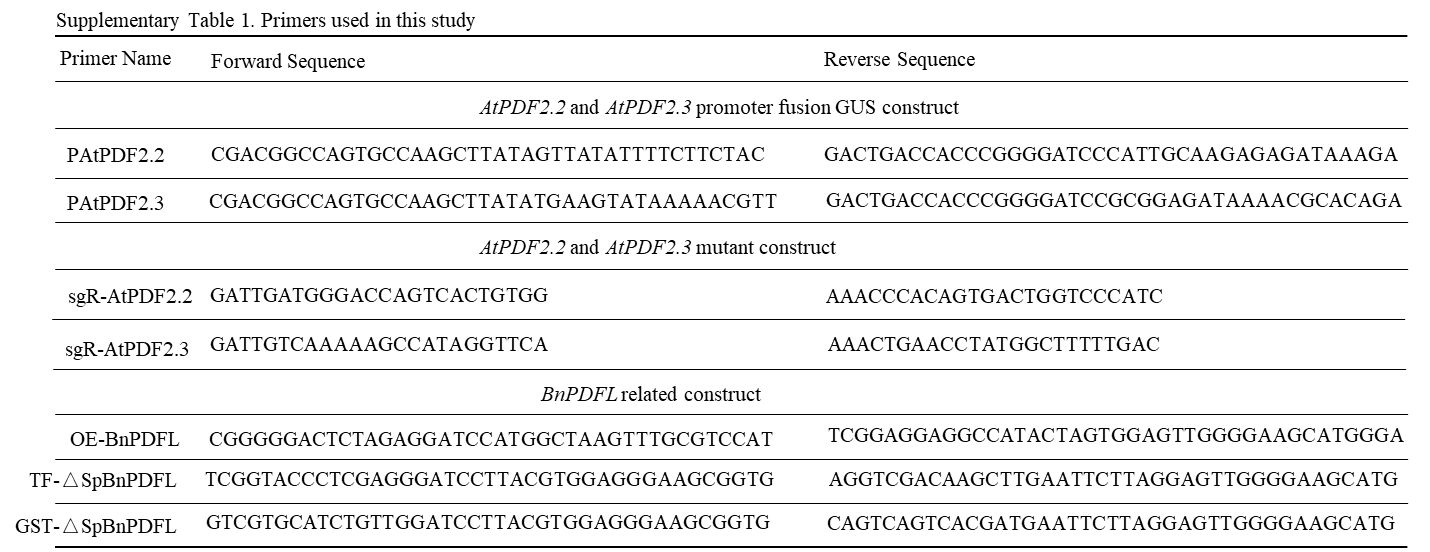

Supplement: Supplementary file 8 — Table S1. Primers used in this study. (DOCX 104 kb) [file 12870_2019_1895_MOESM8_ESM.docx]
