# Supplementary material for: Proteomic changes in the xylem sap of Brassica napus under cadmium stress and functional validation
Source: BMC Plant Biol. 2019 Jun 26;19:280. doi: 10.1186/s12870-019-1895-7 (PMC6595625; doi:10.1186/s12870-019-1895-7)
Supplement: Supplementary file 2 — Figure S2. Subcellular localization prediction of the identified proteins. (DOCX 32 kb) [file 12870_2019_1895_MOESM2_ESM.docx]

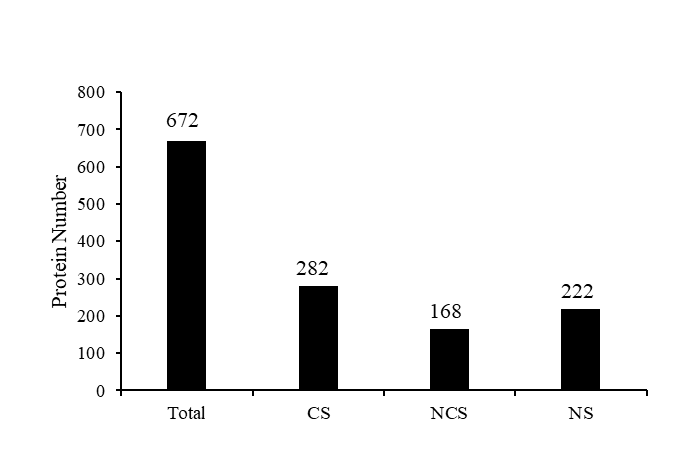


Additional file 2: **Figure S2.** Subcellular localization prediction of the identified proteins

CS = Classical secretory proteins, NCS = non-classical secretory proteins, NS = non-secretory proteins
