# Supplementary material for: Proteomic changes in the xylem sap of Brassica napus under cadmium stress and functional validation
Source: BMC Plant Biol. 2019 Jun 26;19:280. doi: 10.1186/s12870-019-1895-7 (PMC6595625; doi:10.1186/s12870-019-1895-7)
Supplement: Supplementary file 3 — Figure S3. Numbers of identified proteins in the control and Cd-treated samples. (DOCX 171 kb) [file 12870_2019_1895_MOESM3_ESM.docx]

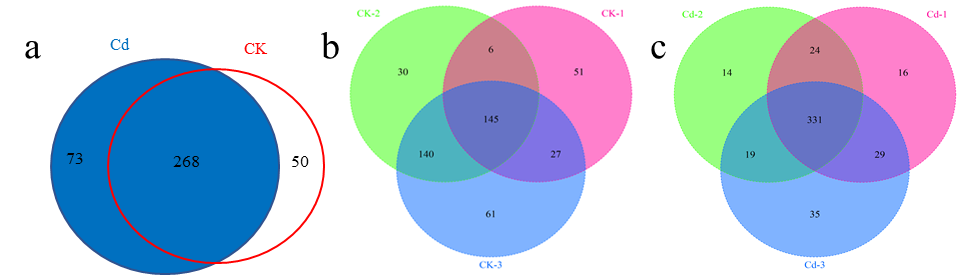


Additional file 3: **Figure S3.** Numbers of identified proteins in the control and Cd-treated samples.

Venn diagram of the number of proteins (**a**) identified in (**b**) the control and (**c**) Cd-treated xylem sap samples of hydroponic-grown *Brassica napus.*
