# Supplementary material for: Proteomic changes in the xylem sap of Brassica napus under cadmium stress and functional validation
Source: BMC Plant Biol. 2019 Jun 26;19:280. doi: 10.1186/s12870-019-1895-7 (PMC6595625; doi:10.1186/s12870-019-1895-7)
Supplement: Supplementary file 4 — Figure S4. Basic characteristics of proteins identified in Brassica napus xylem sap. (DOCX 391 kb) [file 12870_2019_1895_MOESM4_ESM.docx]

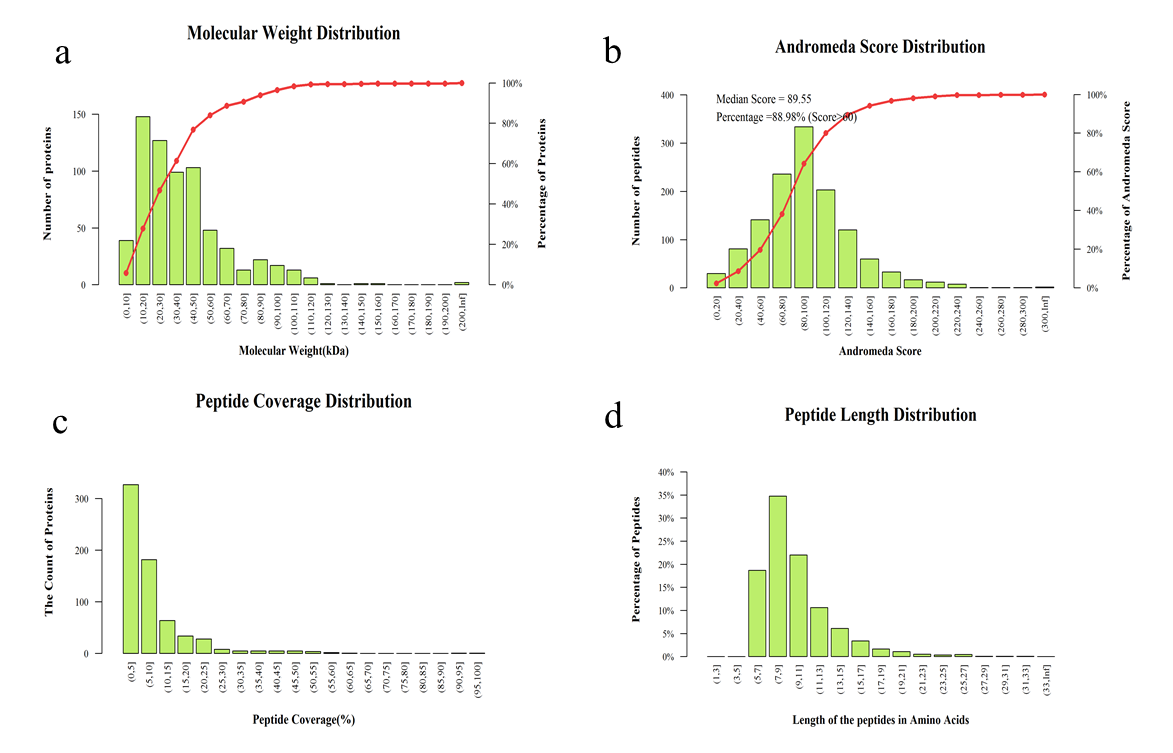


Additional file 4: **Figure S4.** Basic characteristics of proteins identified in *Brassica napus* xylem sap.

Distribution of (**a**) molecular weight, (**b**) andromeda score, (**c**) peptide coverage, and (**d**) peptide length, for proteins in the xylem sap of hydroponics-grown *Brassica napus* identified using a shotgun LC-MS/MS approach.
