# Supplementary material for: Proteomic changes in the xylem sap of Brassica napus under cadmium stress and functional validation
Source: BMC Plant Biol. 2019 Jun 26;19:280. doi: 10.1186/s12870-019-1895-7 (PMC6595625; doi:10.1186/s12870-019-1895-7)
Supplement: Supplementary file 5 — Figure S5. Bioinformatics analysis of BnPDFL. (DOCX 199 kb) [file 12870_2019_1895_MOESM5_ESM.docx]

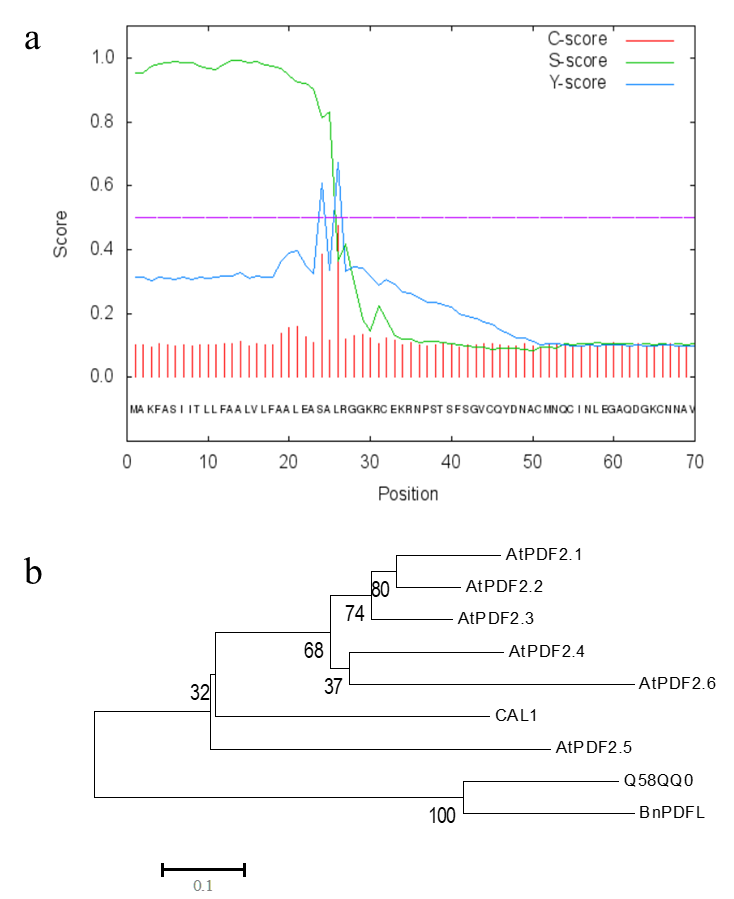


Additional file 5: **Figure S5**. Bioinformatics analysis of BnPDFL. (**a**) *BnPDFL* secretion signal peptide predication by web tool SignaIP 4.0 server. The signal peptide prediction cleavage site is between position 25 and 26 amino acids. (**b**) Phylogenetic relationship of defensin proteins in rice (CAL1), *Arabidopsis thaliana* (AtPDF2s) and *Brassica napus* (BnPDFL, Q58QQ0). The 0.1 scale shows substitution distance.
