# Supplementary material for: Proteomic changes in the xylem sap of Brassica napus under cadmium stress and functional validation
Source: BMC Plant Biol. 2019 Jun 26;19:280. doi: 10.1186/s12870-019-1895-7 (PMC6595625; doi:10.1186/s12870-019-1895-7)
Supplement: Supplementary file 6 — Figure S6. BnPDFL is a Cell wall localized protein (DOCX 1838 kb) [file 12870_2019_1895_MOESM6_ESM.docx]

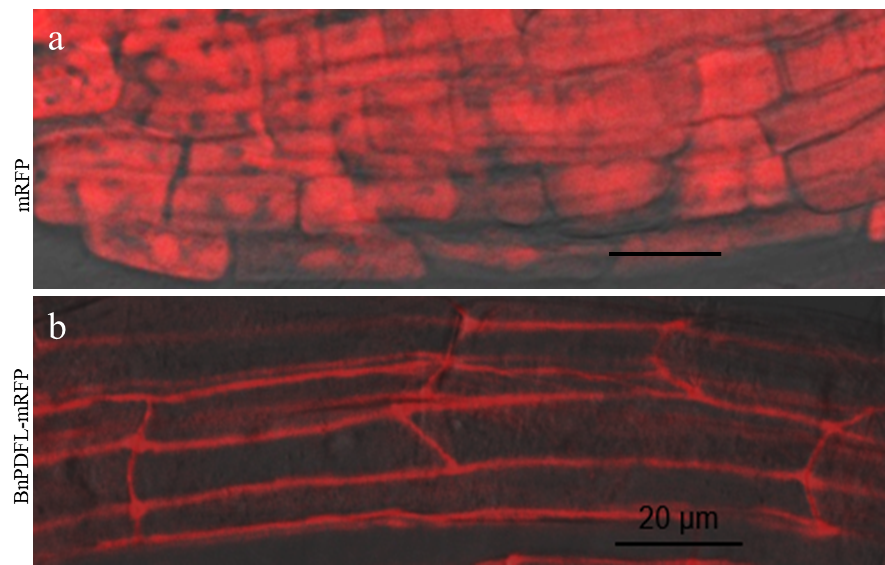


Additional file 6: **Figure S6.** BnPDFL is a Cell wall localized protein

Subcellular localization of mRFP control or BnPDFL in *Arabidopsis* plants harboring the construct 35S::mRFP (**a**) or 35S::BnPDFL -mRFP (**b**). Root epidermal cells were incubated in 40% sucrose to induce plasmolysis and then imaged by confocal microscopy.
