# Supplementary material for: Proteomic changes in the xylem sap of Brassica napus under cadmium stress and functional validation
Source: BMC Plant Biol. 2019 Jun 26;19:280. doi: 10.1186/s12870-019-1895-7 (PMC6595625; doi:10.1186/s12870-019-1895-7)
Supplement: Supplementary file 7 — Figure S7. Histochemical localization of GUS activity in transgenic plants expressing the GUS reporter gene under the control of the proAtPDF2.3 or proAtPDF2.2 promoter. (DOCX 1753 kb) [file 12870_2019_1895_MOESM7_ESM.docx]

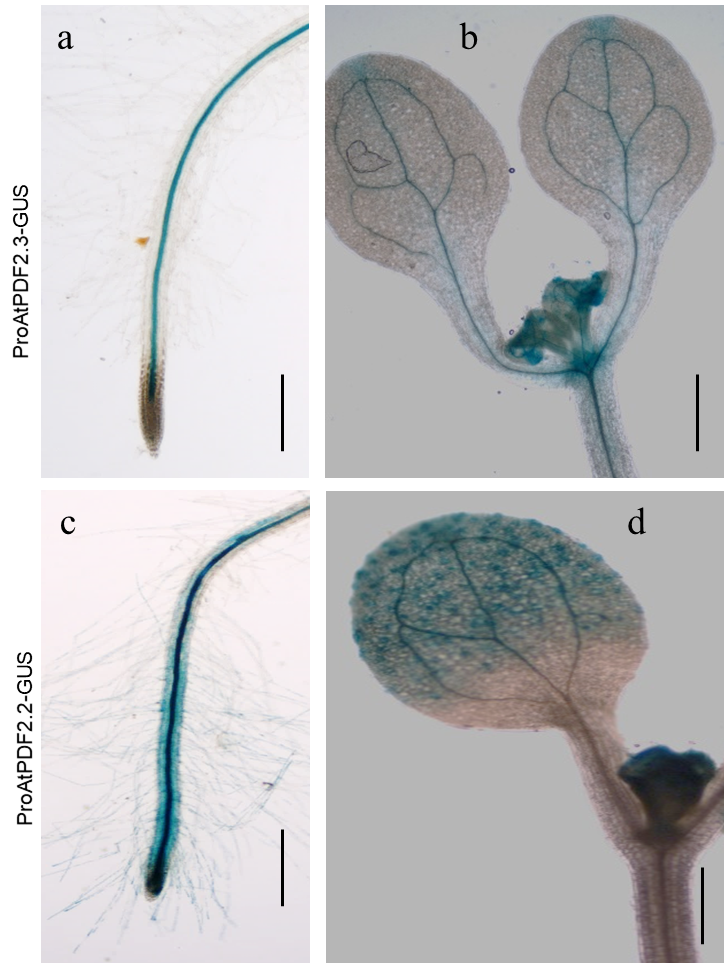


Additional file 7: **Figure S7.** Histochemical localization of GUS activity in transgenic plants expressing the GUS reporter gene under the control of the proAtPDF2.3 or proAtPDF2.2 promoter.

(**a**-**b**) One week old whole-mount ProAtPDF2.3-GUS transgenic seedling root (**a**) and shoot (**b**).

(**c**-**d**) One week old whole-mount ProAtPDF2.2-GUS transgenic seedling root (**c**) and shoot (**d**).

Scale bar = 1mm in (**a**, **c**) or 5mm in (**b**, **d**).
